# Investigation of Fracture Characteristics and Energy Evolution Laws of Model Tunnels with Different Shapes Subjected to Impact Load

**DOI:** 10.3390/ma18040889

**Published:** 2025-02-18

**Authors:** Fukuan Nie, Xuepeng Zhang, Lei Zhou, Haohan Wang, Jian Hua, Bang Liu, Bo Feng

**Affiliations:** 1 State Key Laboratory of Intelligent Construction and Healthy Operation and Maintenance of Deep Underground Engineering, College of Architecture and Environment, Sichuan University, Chengdu 610065, China; niefukuan1023@163.com (F.N.); rrw_fhhllglm@126.com (H.W.); hua_jian0928@163.com (J.H.); 2 State Key Laboratory of Strata Intelligent Control and Green Mining Co-Founded by Shandong Province and the Ministry of Science and Technology, Shandong University of Science and Technology, Qingdao 266590, China; 3 School of Mechanical and Electrical Engineering, Yibin University, Yibin 644000, China; 2014114001@yibinu.edu.cn; 4 China MCC5 Group Corp, Ltd., Chengdu 610063, China; fengbo19910505@163.com

**Keywords:** different shapes of tunnel, dynamic mechanical properties, dynamic stress concentration factor, energy analysis, failure behavior

## Abstract

To investigate dynamic fracture characteristics and failure behavior of different sections of tunnel surrounding rock mass, six kinds of model tunnels were fabricated using green sandstone, and impact tests were performed using a split Hopkinson pressure bar system. The dynamic compressive strength and energy change behaviors of samples comprising different-shaped tunnels were assessed, and crack propagation paths were analyzed employing a digital image correlation method. Numerical calculations were carried out using the software LS-DYNA (v. 2021R1), and the dynamic stress concentration factors of different model tunnel samples were determined. The results of the research indicated that the shape of the tunnel affected the dynamic compressive strength. The elliptical tunnel had the smallest percentage of dissipated energy, and the three-centered circular tunnel had the largest percentage of dissipated energy. The maximum tensile stress concentration factor in the model tunnels consistently occurred at the top or bottom; so, the locations of initiation were most commonly at the bottoms and tops of the tunnels. Sample failure resulted from a combination of tensile and shear cracks, with the failure mode being primarily tensile-dominated. Finally, the inverted arch had an obvious alleviating action on the stress concentration phenomenon at the bottom of the three-centered circle.

## 1. Introduction

In underground engineering practices, tunnel structures are often subjected to various dynamic loads during excavation or usage. For instance, during mining, oil extraction, or tunnel construction, dynamic loads may arise from factors such as impacts, explosions, blasting, drilling, and seismic activity. These dynamic loads can significantly affect the safety and stability of tunnel structures [1,2,3]. The stress concentration and redistribution induced by the action of dynamic load [4,5] can lead to various types of rock collapse, often occurring near openings. Rock instability in underground projects leads to significant economic losses and presents a serious risk to the safety of workers. Thus, gaining a thorough understanding of the failure and fracture behavior of tunnel rock under dynamic load is crucial for safeguarding the integrity of underground construction.

In recent years, researchers have made significant achievements in the mechanical behavior and fracture characteristics of tunnel rock bodies subjected to dynamic loading. Tan et al. [6] performed a series of experiments on a single rectangular cavity rock mass and a rectangular cavity rock mass containing two different arrangements, to analyze the dynamic response and fracture evolution behavior of the rock mass containing cavities. Tao et al. [7] investigated the failure mechanism of laminar cracking of rock samples with elliptical holes subjected to dynamic stress waves. They proposed that the amplitude and wavelength of the stress waves greatly affected the distribution of the dynamic stress concentration around the elliptical holes, which in turn affected the rupture mode of the rock samples. Wu et al. [8] conducted a study on the dynamic mechanical response and energy dissipation of rock samples with different sizes of circular holes and concluded that the introduction of holes led to a significant reduction in the overall mechanical performance of the rock. Liu et al. [9] experimentally investigated dynamic and static joint impact on shale samples containing circular holes with different lamination angles. They highlighted that the primary cause of initial cracking was the stress concentration induced by the circular holes. Therefore, investigating the failure modes of a tunnel under dynamic load is of paramount importance. However, most of the above studies have focused on the fracture process of tunnel samples, while analysis of energy and dynamic stress concentration factors is still relatively lacking.

Furthermore, numerical simulations have emerged as a key approach for investigating the failure behavior of rocks subjected to dynamic load conditions [10,11,12]. Zhou et al. [13] used AUTODYN code to simulate the crack propagation paths and analyze the fracture mechanism of a cracked tunnel. Qiu et al. [14] applied PFC2D to simulate the dynamic response characteristics of inverted U-shaped deeply buried tunnels under explosive loading, taking into account the effects of blast orientation, the distance between the source of the blast and the tunnel, and the depth of burial. You et al. [15] simulated the progressive cracking of single-defect samples under different axial prestress constraints using the discrete element method. Rodríguez et al. [16] proposed a finite element method to estimate the elastic settlement of foundations for tunnels excavated in porous granular media on strata assumed to be completely impermeable, providing important insights into settlement prediction related to tunnel excavation.

In previous studies, the effects of factors such as tunnel size, dynamic loading direction, and dynamic loading magnitude have been discussed. This has deepened the understanding of tunnel mechanisms and provided a basis for safe design guidelines to minimize damage and destruction problems in the tunnel rock mass. Furthermore, the effect of tunnel shape on the failure mode under static load has been extensively studied by numerous researchers [17,18,19]. For example, Zhang et al. [18] and Zeng et al. [19] experimentally studied the mechanical properties and cracking behavior of specimens with different shapes of holes under uniaxial compression load. However, there has been little discussion on the shape of the tunnel in the rock under dynamic load, which is an equally important issue for tunnel engineering. Therefore, this study attempted to explore the effect of tunnel shape under dynamic load to better understand the cracking mechanism of the rock mass and to provide a more solid foundation for the safe design of the rock mass. Different shapes of tunnel samples were made using green sandstone, and dynamic loading was applied using a split Hopkinson pressure bar (SHPB) apparatus. The dynamic fracture process, crack propagation patterns, and energy evolution characteristics of the tunnel model were monitored using a high-speed camera and analyzed through the digital image correlation (DIC) technique. Finally, finite element software (LS-DYNA) was used to simulate the failure process of the tunnel. Based on the experimental results, valuable insights into underground excavation are presented, which can be used to provide theoretical guidance for tunnel stability under dynamic impact.

## 2. Research Methodology

**Figure 1:**
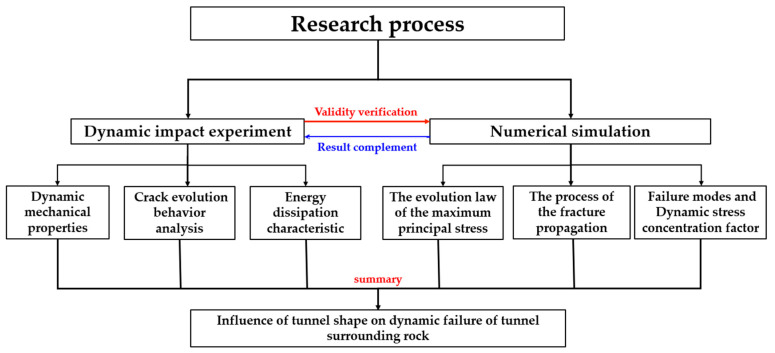
Research flow chart.

In this study, a combination of experimental and numerical simulation was used; the specific process is shown in Figure 1.

### 2.1. Specimen Preparation

Green sandstone is a crushed feldspar schist with large amounts of pyroxene. It is sedimentary and consists mainly of quartz, feldspar, clasts, and heavy minerals with good chemical stability. Since it is mainly composed of cemented and accumulated sand grains and is dense and homogeneous, relatively structurally stable, and easy to process, it is often used for laboratory tests [20]. It has a certain superiority for idealizing various types of tunnel model samples in a homogeneous mechanical model of a continuous medium. The mechanical parameters of the green sandstone used in this study are listed in Table 1.

**Figure 2:**
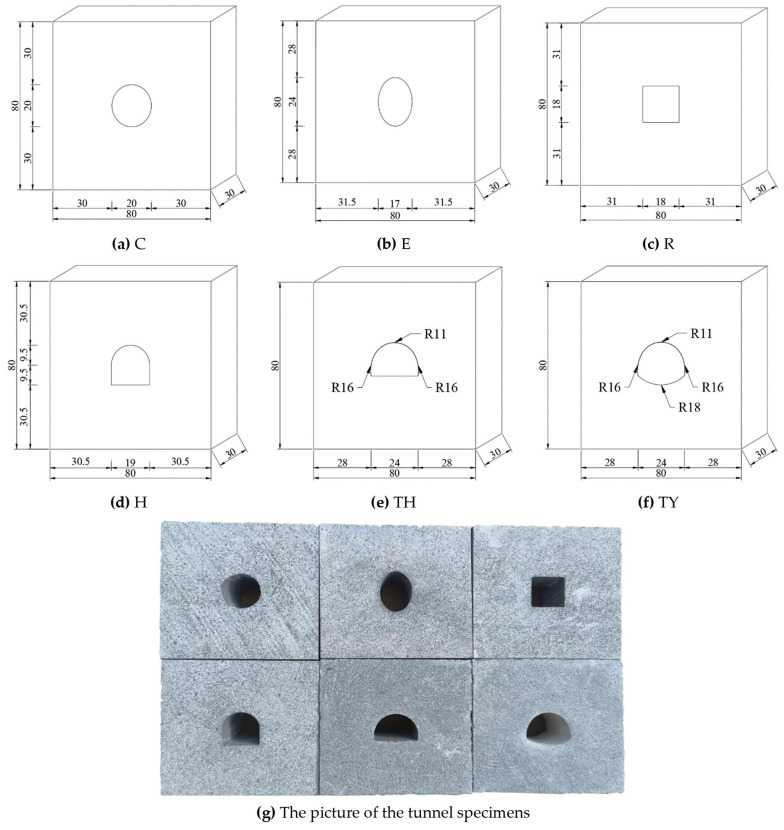
Sketch map of the tunnel specimens (unit: mm).

To investigate the fracture behavior of the tunnel models, six types of tunnels, namely a circular tunnel (C), elliptical tunnel (E), rectangular tunnel (R), horseshoe-shaped tunnel (H), three-centered circular tunnel (TH), and three-centered circular tunnel with inverted arch (TY), were chosen for this study. Among these, the three-centered circular tunnel and three-centered circular tunnel with an inverted arch were designed on an equal scale according to the Chinese “Specifications for Design of Highway Tunnels” [21]. To minimize the influence of thickness, all tunnels were positioned as centrally as possible within the samples, considering the diameter of the SHPB. To study the effect of the shape of the tunnels, the cross-sectional areas of the tunnels were made identical. The dimensions of all tunnel model samples are shown in Figure 2. The sample size was 80 mm × 80 mm × 30 mm (length × width × thickness), and each group of five samples totaled 30 samples. The samples were made of sandstone slate with good texture and integrity, with no macroscopic defects visible to the naked eye, such as laminations, cracks, weak interlayers, etc. The slate was first cut, then perforated with a high-pressure water jet cutter, and the sides and ends of the samples were polished and smoothed. Before the dynamic fracture experiment, all samples were thoroughly cleaned and coated with a layer of white paint. Once the white paint had dried, black dots were applied to create randomly distributed scattered regions on the surface.

### 2.2. Experimental Protocol

**Figure 3:**
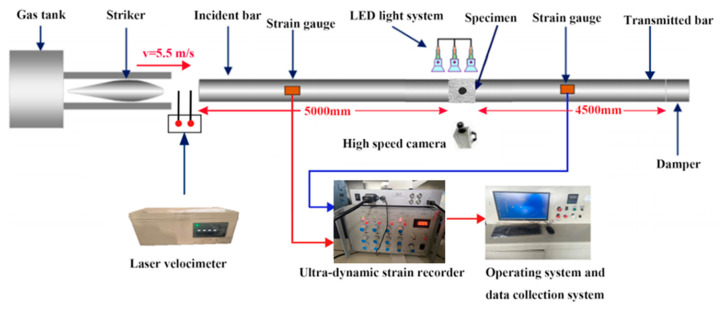
SHPB test system.

To simulate dynamic loading, an impact test was carried out using a SHPB device, as shown in Figure 3. The bars are constructed from CrMnSiA steel with a diameter of 100 mm. The material properties of the bars included Young’s modulus of 210 GPa, Poisson’s ratio of 0.28, and a density of 7850 kg/m^3^. The incident bar is 5000 mm, and the transmission bar is 4500 mm. The striker was a spindle with a length of 400 mm, which produced a relatively smooth half-sinusoidal stress wave curve [22]. To minimize friction effects, a thin layer of butter was applied to both ends of the sample before each test. The pressure was set to 0.075 MPa, corresponding to an impact velocity of 5.5 m/s for the striker. A copper sheet with a diameter of 24 mm and a thickness of 1 mm was affixed to the incident end of the incident bar to mitigate the scattering effect of the incident wave.

### 2.3. Validity of SHPB

According to the one-dimensional stress wave theory, the dynamic stress *σ*(*t*), strain *ε*(*t*), and strain rate $\dot{\varepsilon }$(*t*) were calculated as follows [23]:

(1) $$\sigma (t)=\frac{A_{e}E_{e}}{2A_{s}}\left[\varepsilon_{i}\left(t\right)+\varepsilon_{r}\left(t\right)+\varepsilon_{t}\left(t\right)\right]$$

(2) $$\varepsilon (t)=\frac{C}{L_{s}}\int_{0}^{t}\left[-\varepsilon_{i}\left(t\right)+\varepsilon_{r}\left(t\right)+\varepsilon_{t}\left(t\right)\right]dt$$

(3) $$\dot{\varepsilon }(t)=\frac{C_{0}}{L_{s}}\left[-\varepsilon_{i}\left(t\right)+\varepsilon_{r}\left(t\right)+\varepsilon_{t}\left(t\right)\right]$$

where *A*_e_ and *E*_e_ are the cross-sectional area and elastic modulus of the bars, respectively; *A*_s_ and *L*_s_ are the cross-sectional area and length of the tunnel specimens, respectively; *ε*_i_(*t*), *ε*_r_(*t*), and *ε*_t_(*t*) are the incident strain signal, the reflected strain signal, and the transmitted strain signal, respectively; and *C*_0_ is the longitudinal wave velocity of the bars.

**Figure 4:**
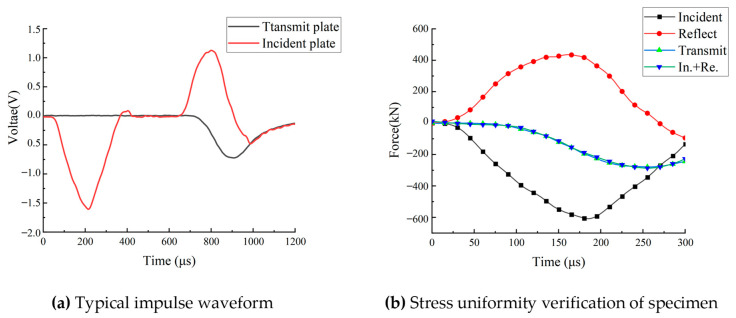
Dynamic loadtime history of the tunnel specimen.

The SHPB test system should satisfy the one-dimensional plane stress wave theory. Figure 4 shows the dynamic loading process as well as the stress history. The superposition curves of the incident and reflected waves align with the time-distance curves of the transmitted waves, demonstrating that the tunnel specimen met the stress equilibrium condition under dynamic loading, validating the reliability of the SHPB test in this study.

### 2.4. Digital Image Correlation Technology

**Figure 5:**
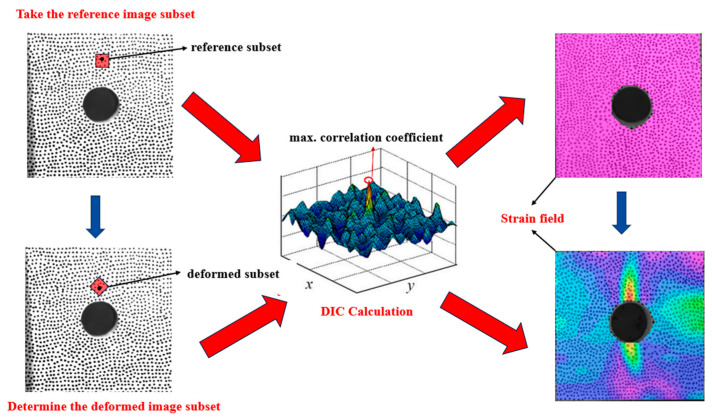
The principle of the DIC method.

Digital image correlation (DIC) technology has been widely used for studying the fracture behavior of rock samples and plays an important role in analyzing the deformation and rupture laws of pore-containing rocks under dynamic load [24,25,26,27]. By comparing the displacement variations between the reference and target points during the loading process, DIC technology effectively tracks the dynamic evolution of the sample’s displacement field. From this, the strain field can be derived, as illustrated in Figure 5. The crack propagation law and fracture characteristics of the different model tunnel samples were investigated using DIC technology, aiming to reveal the impact of various tunnel shapes on the failure mode of the rock and to provide a theoretical basis for stability analysis and design of rock engineering.

### 2.5. Numerical Simulation Method

#### 2.5.1. Material Modeling

**Figure 6:**
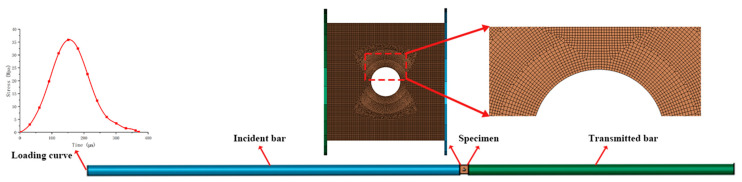
Numerical model.

To explore the fracture behavior of cracks in different shapes of tunnels, numerical simulations were carried out using LS-DYNA software. Figure 6 illustrates the three-dimensional finite element model, which closely resembles the impact fracture test. The model was built with solid elements, and the numerical simulation employed the Lagrangian approach for calculation. For a circular tunnel model, a total of 1,140,465 elements and 1,192,434 nodes were meshed. The contact between the incident bar, the green sandstone sample, and the transmitted bar was defined using *CONTACT_AUTOMATIC_SURFACE_TO_SURFACE, with a friction coefficient of 0 and a contact penalty coefficient of 2. Dynamic stress waves were applied to one side of the incident bar during the test.

The selection of the sample material model is key to the success of numerical simulation. LS-DYNA software offers a variety of material models suitable for simulating rock behavior; the most commonly used of these are the Cowper–Symonds model (PK), the JOHNSON_HOLMQUIST_CERAMICS model (JH-2), the Continuous Surface Caps Model (CSCM), and the Riedel–Hiermaier–Thoma (RHT) model [28]. Generally, the PK model lacks sufficient material parameters to effectively capture the complex dynamic response of rock under dynamic loading, while the JH-2 model is insufficient for accurately describing the tensile failure of rock and the CSCM model is too complicated in terms of parameter determination, and this has a general effect in practical application. The RHT model comprehensively considers the strain rate effect, the effect of surrounding pressure, the effect of strain hardening, and the effect of damage softening, while at the same time considering the compression damage and tensile damage caused to the materials. In summary, compared with several other materials, the RHT model is capable of precisely representing the fracture characteristics of rock under dynamic load [29].

There are many parameters to be examined in the RHT model, and these can be roughly classified into seven types [30]. While some of these parameters can be determined through experimental and theoretical methods, others are more complex to obtain and must be sourced from the existing literature. The RHT model for green sandstone, described by Li et al. [31], was applied in this study. The simulation results obtained were close to the experimental results, proving the set of parameters to be reasonable. The corresponding RHT parameters for green sandstone are provided in Table 2.

#### 2.5.2. Calculation of Dynamic Stress Concentration Factor

**Figure 7:**
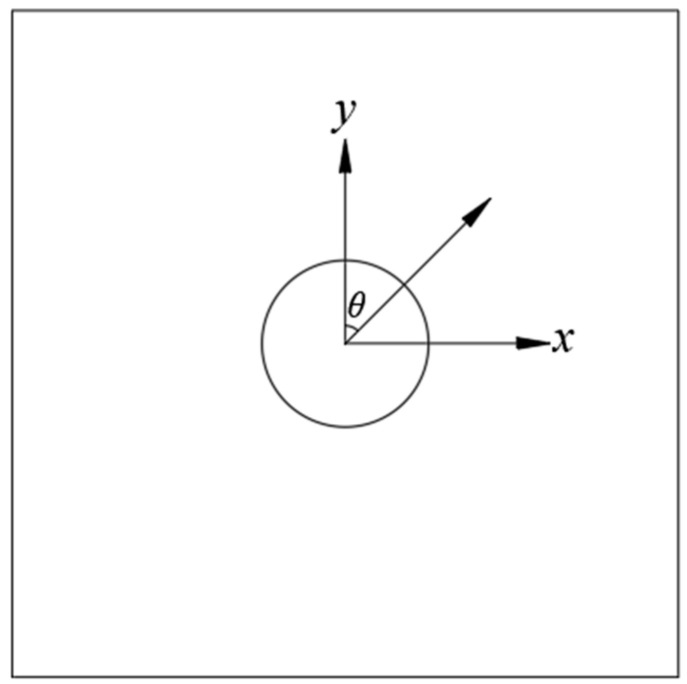
Schematic of the polar coordinate system.

The stress concentration phenomenon is particularly significant in tunnels with isotropic variations, especially in regions of isotropic variations at the tunnel corners. To better understand the stress concentration phenomenon around the tunnels under dynamic load, the dynamic stress concentration factor (DSCF) around the tunnels was calculated. To study and analyze the radial and circumferential stress distribution around each tunnel, and to simplify the analysis, the center of the sample (coinciding with the tunnel’s center) was taken as the origin of the polar coordinate system. The coordinate system was constructed as shown in Figure 7, with the polar angle θ incremented in the clockwise direction. Using the following equations, the radial and circumferential stresses at any point of the tunnel envelope can be derived [32]:

(4) $$\sigma_{\theta \theta }=\sigma_{x}\cos^{2}\theta +\sigma_{y}\sin^{2}\theta -2\tau_{xy}\sin\theta \cos\theta$$

(5) $$\sigma_{rr}=\sigma_{x}\sin^{2}\theta +\sigma_{y}\cos^{2}\theta +2\tau_{xy}\sin\theta \cos\theta$$

where *σ*_θθ_ is the circumferential tensile stress; σ_rr_ denotes the radial tensile stress; and *σ*_x_, *σ*_y_, and *τ*_xy_ are the horizontal, vertical, and shear stresses, respectively, of the monitoring unit in the plane coordinate system.

When the stress wave impinges on the tunnel, the failure of the tunnel’s perimeter is strongly influenced by the distribution of *σ*_θθ_. So, this study focused on the distribution of *σ*_θθ_ around the tunnel in different tunnel shapes under dynamic load. The DSCF was defined as the maximum *σ*_θθ_ divided by the amplitude of the incident stress wave at the same location when the tunnel was not included. The negative value of the DSCF represents the tensile stress concentration. When the tensile stress is concentrated, the smaller the value of the DSCF, the greater the degree of tensile stress concentration.

## 3. Results

### 3.1. Eperimental Results

#### 3.1.1. Dynamic Mechanical Properties

**Figure 8:**
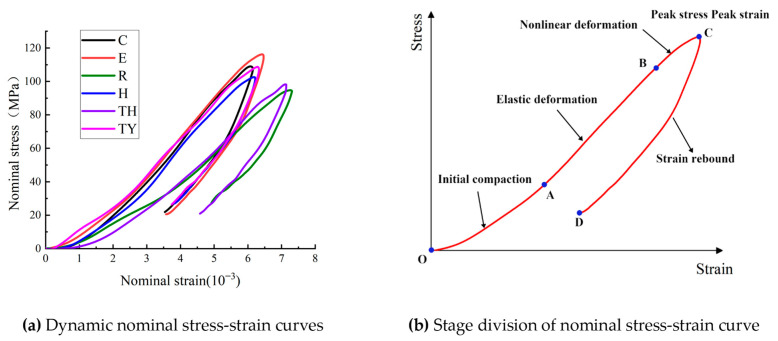
Dynamic nominal stress strain curves of some specimens.

In the following, the relative deformation and the corresponding stress of the green sandstone tunnel samples are defined as “nominal strain” and “nominal stress,” respectively. Figure 8 shows the nominal stress–strain curves of some typical samples. The process can be broadly classified into four distinct stages: (1) compaction (OA), where microcracks and voids are compressed; (2) elastic deformation (AB), during which the sample’s nominal stress increases approximately linearly with nominal strain, and the dynamic stress–strain curve exhibits elastic growth within a specific range; (3) plastic deformation (BC), characterized by a deviation from linearity in the nominal stress–strain curve. Prior to reaching the peak nominal stress, the increase in stress slows down relative to strain, and the slope of the curve gradually decreases. During this phase, microcracks initiate, nucleate, and propagate due to localized stress concentrations around the tunnels [33]; (4) failure (CD), where nominal stress significantly decreases after it reaches the maximum point, and the curve slope becomes negative. The dynamic nominal stress–strain curve exhibits significant hysteresis. This is attributed to the release of accumulated elastic strain energy during the post-peak phase, which corresponds to the unloading section of the curve. Despite complete unloading, permanent deformation persists due to the formation and growth of microcracks throughout loading. In this final stage, further crack propagation leads to the failure of the model tunnel sample.

**Figure 9:**
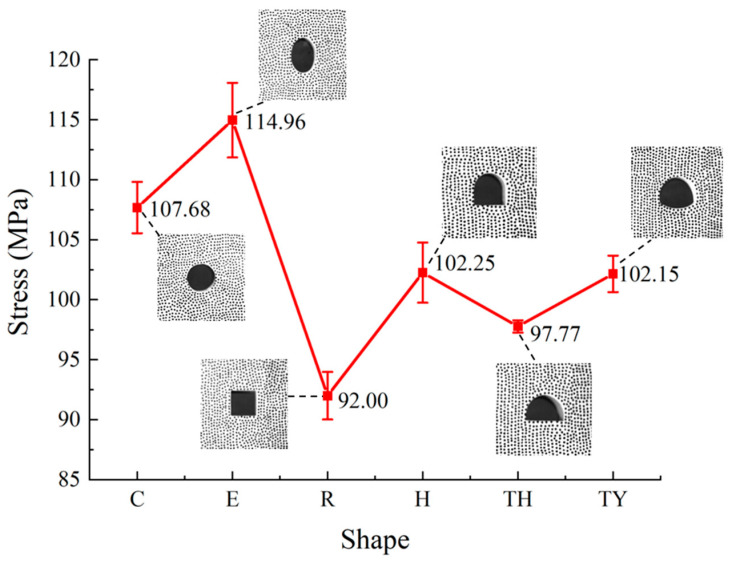
The average dynamic compressive strength of the model tunnels with different shapes.

Figure 9 demonstrates the difference in dynamic compressive strength (DCS) between the six tunnel types. The samples’ strength values followed the order: E > C > H > TY > TH > R. The elliptical tunnel strength was first of these, at 114.97 MPa, which was 6.34% greater than that of the circular tunnel at 107.68 MPa, since an elliptical tunnel structure can distribute stresses more uniformly when the long axis is stressed, compared with a circular tunnel structure. Wang et al. [34] reported that under the same cross-section conditions when the long axis of the elliptical tunnel was parallel to the disturbing stress, an elliptical tunnel was more resistant to failure than a circular tunnel, which is consistent with the experimental results in this study.

The lower regions of the horseshoe-shaped tunnel and three-centered circular tunnel were easily subject to stress concentration and more likely to be fractured, so their strength was lower than that of the circular tunnel and the elliptical tunnel. DCS has a positive linear relationship with the amplitude of the transmitted wave. In the case of similar shapes, as the length of the tunnel boundary perpendicular to the direction of impact increases, the amplitude of the transmitted stress wave decreases, resulting in a reduction in the sample’s DCS. This relationship provides a clear explanation for why the DCS of the horseshoe-shaped tunnel was higher than that of the three-centered circular tunnel sample, as their vertical boundary lengths were 19 mm and 24 mm, respectively. The DCS of the three-centered circular tunnel with an inverted arch was higher than that of the three-centered circular tunnel because the inverted arch relieved the stress concentration phenomenon at the bottom of the three-center circle tunnel, thereby improving the strength of the tunnel. The most prone to failure was the rectangular tunnel, which was prone to stress concentration at the four corners of the rectangular tunnel, which in turn affected its strength. Under static load, Wu et al. [17] also described the compressive strength of circular tunnel > horseshoe-shaped tunnel > rectangular tunnel for several shapes, consistent with the law of DCS. Overall, the shape of the tunnel affected the DCS of the model tunnel samples.

#### 3.1.2. Crack Evolution Behavior Analysis

**Figure 10:**
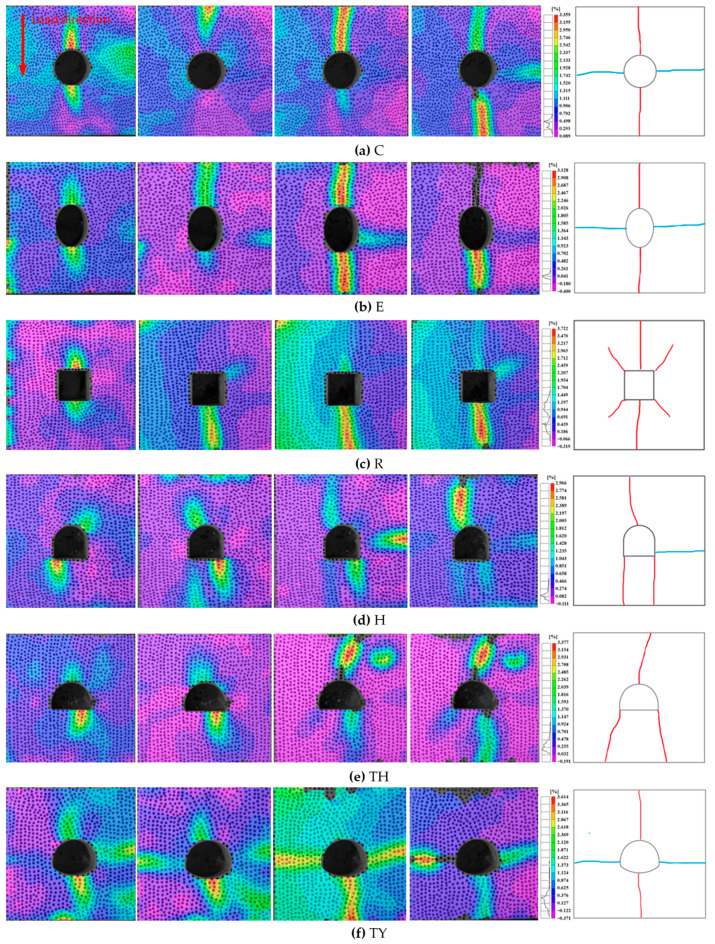
Fracture evolution of tunnel specimens of different shapes. (red represents the main crack and blue represents the secondary crack.).

This study investigated the crack evolution behavior of different tunnel shapes under dynamic stress waves, including the location of initiation and the propagation process. A high-speed camera was used to record the fracture process. The captured images were then subjected to DIC processing to derive the strain distribution of the tunnel samples with different shapes, as shown in Figure 10. The main crack in the circular tunnel sprouted from the top and bottom ends and propagated toward the outer end of the sample along the loading direction and through to the sample’s end. The crack propagation in the elliptical tunnel was similar to that in the circular tunnel. In the rectangular tunnel, in addition to the main cracks at the top and bottom ends, cracks were randomly generated at the four corners. In the horseshoe-shaped tunnel, cracks were generated at the top of the arch and the two lower corners and propagated toward the edges. In contrast to the horseshoe-shaped tunnel, the three-centered circular tunnel had cracks at the bottom center of the tunnel in addition to cracks at the bottom corners, and the cracks propagated outward. Finally, in the three-centered circular tunnel with an inverted arch, cracks propagated from the top and bottom ends of the tunnel until they penetrated through the sample, and cracks were not generated at the bottom corners, suggesting that the inverted arch improved the stability of the tunnel. The secondary cracks in all the samples were randomly generated at the left and right ends of the samples after the primary cracks were generated and propagated from the edges of the samples into the tunnels, as shown in Figure 10. This was due to the effect of multiple impacts on the samples when the waves were reflected many times and repeatedly transmitted to the samples.

#### 3.1.3. Energy Dissipation Characteristic

Based on the energy conservation equation, the relationship between incident energy *W*_i_, reflected energy *W*_r_, transmitted energy *W*_t_, and dissipated energy *W*_s_ in the SHPB testing system was derived. The formula for calculating the dissipated energy *W*_s_ is as follows:

(6) $$W_{i}=A_{e}CE_{e}\int_{0}^{t}\varepsilon_{i}^{2}\left(t\right)dt$$

(7) $$W_{r}=A_{e}CE_{e}\int_{0}^{t}\varepsilon_{r}^{2}\left(t\right)dt$$

(8) $$W_{t}=A_{e}CE_{e}\int_{0}^{t}\varepsilon_{t}^{2}\left(t\right)dt$$

(9) $$W_{s}=W_{i}-W_{r}-W_{t}$$

With the same impact air pressure, the incident energy of the green sandstone model tunnel samples ranged from 162 to 203 J. The amount of inflation may have been slightly different each time, and the interval between starting the vent and triggering the firing button was different each time, resulting in slightly different impact velocities of the striker bar, which led to slightly different incident energy. The fragmentation characteristics of the tunnel samples under dynamic load were simplified and analyzed via a series of assumptions. First, it was assumed that the fragments had equal magnitudes of velocity during fragmentation, to help ignore complex interactions caused by velocity differences between fragments. Second, it was assumed that the samples fragmented uniformly along the radial direction, ignoring the velocity gradient along the radial direction. In addition, the angular velocities of the fragments were ignored in this process because angular velocities have less effect on the overall crushing characteristics. Based on these assumptions that dissipated energy primarily facilitates crack initiation, propagation, and coalescence within the samples, a dissipated energy percentage was introduced to quantify the fragmentation characteristics of the samples. This ratio reflected the ability of the green sandstone tunnel model samples to absorb and dissipate energy during the crushing process. The larger the value of the dissipated energy ratio, the greater the number of cracks generated within the samples. In this study, the reflected energy ratio Pr, the transmitted energy ratio Pt, and the dissipated energy ratio Ps are used, expressed as follows [35]:

(10) $$P_{r}=\frac{W_{r}}{W_{i}}$$

(11) $$P_{t}=\frac{W_{t}}{W_{i}}$$

(12) $$P_{s}=\frac{W_{s}}{W_{i}}$$

**Figure 11:**
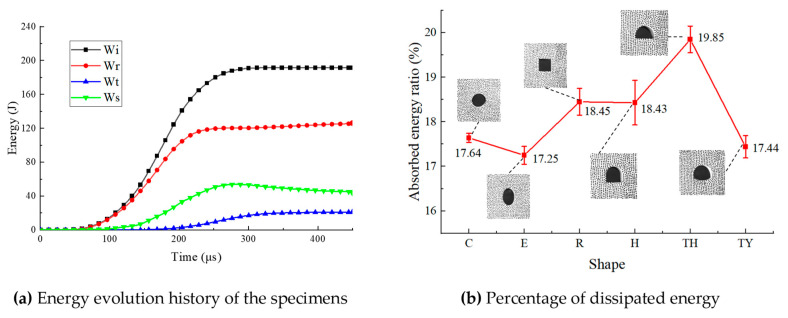
Energy dissipation characteristics.

The energy evolution laws of the tunnel are shown in Figure 11a. A “zero-energy” phase is evident at the start of these curves, as the wave signals in the incident bars must travel a certain distance before being recorded. The transmitted wave signal, after undergoing multiple reflections and transmissions, is captured on the transmitting bar, which results in the “zero-energy” phase of the transmitted energy curve lasting the longest [36]. Figure 11b shows the percentage of dissipated energy for different shapes of tunnels. Macroscopically, the circular tunnel, elliptical tunnel, and three-centered circular tunnel with inverted arch produced two main cracks at the top and bottom, while the horseshoe-shaped tunnel, three-centered circular tunnel, and rectangular tunnel produced three main cracks. Therefore, the dissipated energy ratios of the horseshoe-shaped tunnel, three-centered circular tunnel, and rectangular tunnel were significantly larger than those of the other groups. The transmitted energy relates to the length of the tunnel boundary perpendicular to the impact direction. The proportion of energy transmitted in the three-centered circular tunnel was smaller than in the horseshoe-shaped tunnel; so, the proportion of dissipated energy in the three-centered circular tunnel was larger and the breakage more serious. Similarly, the dissipated energy ratio of the circular tunnel was larger than that of the elliptical tunnel. Since most of the secondary cracks were produced by multiple impacts, that is, after the main cracks were produced, and the dissipated energy ratio calculates the cracking caused by the first impact, the relationship between the number of secondary cracks and the energy was not considered.

### 3.2. Simulation Results

#### 3.2.1. The Evolution Law of Maximum Principal Stress

**Figure 12:**
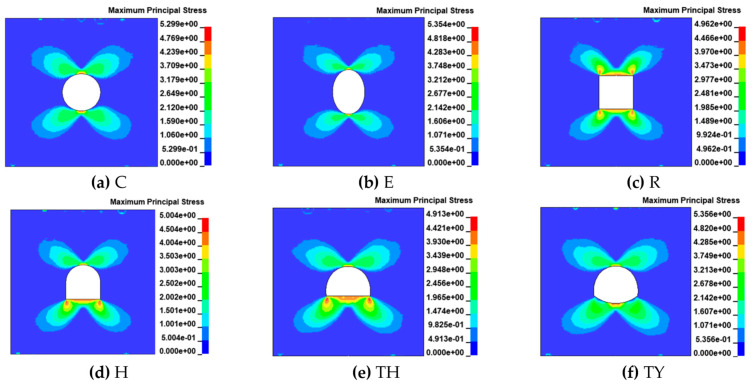
Dynamic maximum principal stress distribution in tunnel rock.

The presence of tunnels as a class of geometrically discontinuous structures changes the state of stresses within the rock mass. During dynamic loading, the initiation and progression of cracks are influenced by the tensile stresses surrounding the tunnel. To understand the crack formation process, it is essential to analyze the stress distribution around the tunnel. Figure 12 shows the distribution of maximum principal stresses (tensile stresses) in various model tunnels at the moment of cracking under dynamic loading. The location of crack initiation is often a region of tensile stress concentration. Ctracks sprout in the rock when the localized tensile stress reaches its maximum tensile strength. In this simulation, the stress wave began to propagate from the left end of the incident bar, at zero time, and the time for the stress wave to reach the tunnel model sample boundary was 802 μs. The graphs of the maximum principal stress distribution in all tunnels were butterfly-shaped, and the maximum distributions of the circles and ellipses were at the top and bottom ends; therefore, the cracks appeared at the top and bottom ends. In the rectangular tunnel, tensile stress was observed to be concentrated at the four corners, except for the stresses at the top and bottom ends, which were relatively high. The horseshoe-shaped tunnel and three-centered circular tunnel similarly showed tensile stress concentration at the top, bottom, and corners, respectively. Compared with the three-centered circular tunnel, the tensile stress at the corners of the three-centered circular tunnel with the inverted arch was obviously much less; so, the cracks in the three-centered circular tunnel with the inverted arch sprouted and propagated only from the tunnel’s top and bottom ends.

#### 3.2.2. The Process of the Fracture Propagation

**Figure 13:**
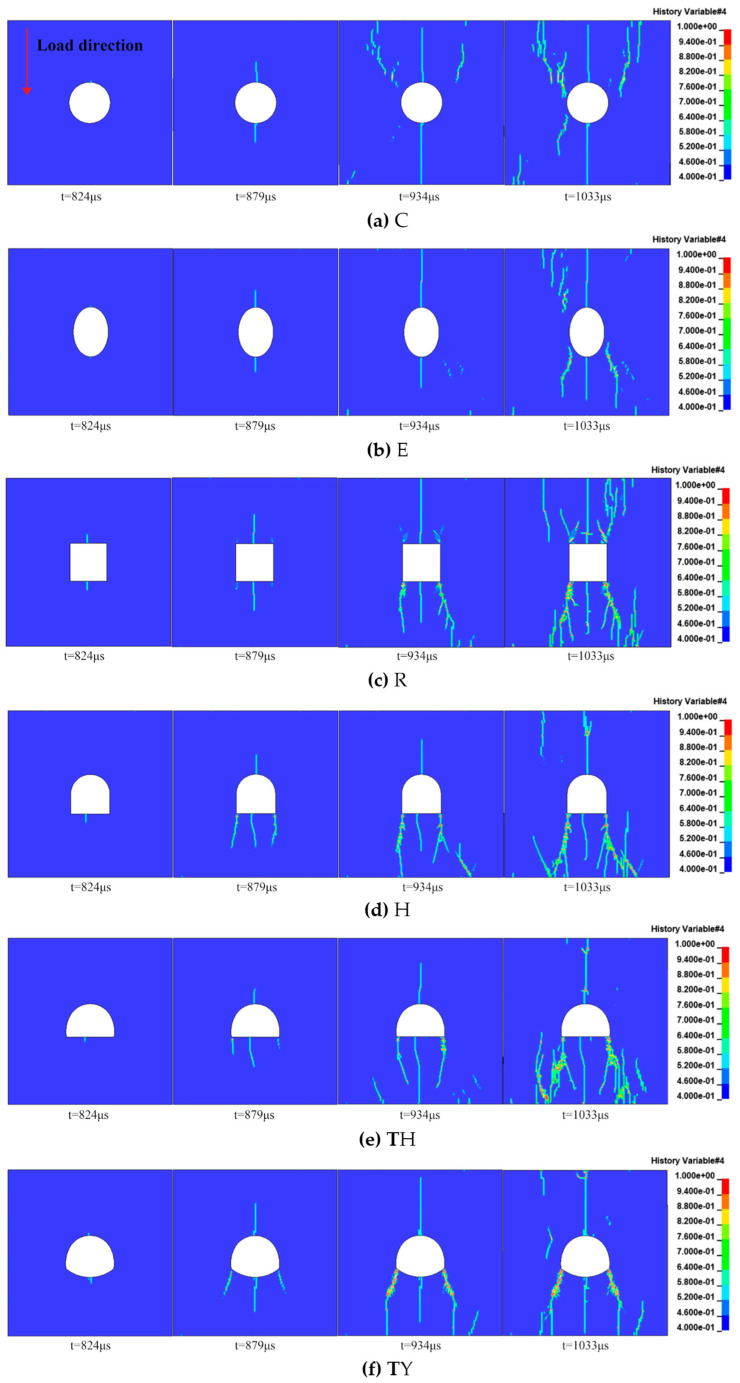
Numerical simulation results of fracture evolution in tunnel specimens of different shapes.

The RHT model applied in the post-processing stage of the simulation was able to detect the degree of damage to the rock. Once the damage value exceeded a certain threshold (0.4 was set in this study), the rock was recognized to have suffered serious failure and cracks. The crack propagation paths versus time are shown in Figure 13. The circular and the elliptical tunnels produced main cracks from the tunnel’s top and bottom ends, and the rectangular first produced main cracks at the top and bottom ends of the tunnel and then at the four corners. The horseshoe-shaped tunnel produced cracks from the top and bottom ends and the lower two corners. The three-centered circular tunnel and the three-centered circular tunnel with an inverted arch both behaved similarly to the horseshoe-shaped tunnel. In all tunnels, after the main cracks were formed, some secondary cracks occurred at the upper and lower ends of the samples and propagated toward the center of the tunnel.

**Figure 14:**
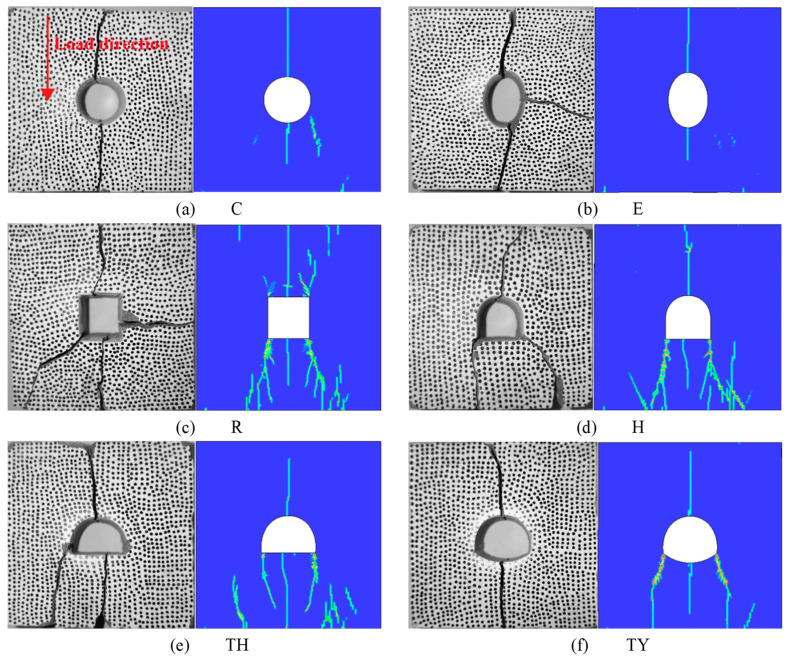
Comparison of experimental and numerical simulation results.

Figure 14 depicts the comparison between the test results and the simulation results, which are in good agreement. The main differences are the following: first, in the rectangular tunnel sample, the numerical simulation produced cracks at all four top corners. However, the test results produced only one random crack at the top corner; secondly, in the horseshoe-shaped tunnel and the three-centered circular tunnel, only two main cracks were produced at the bottom end. The horseshoe-shaped tunnel produced two at the corners at the bottom end, and the three-centered circular tunnel produced one in the middle of the bottom end and one at the corner. The bottom end of the three-centered circular tunnel with an inverted arch produced only one crack. The reason for this phenomenon is that the test impact velocity was not very large, and after the crack was produced, most of the energy had been consumed; so, the crack appeared at the bottom end where it was most likely to be produced. In the simulation, some secondary cracks were produced at the top and bottom ends of the samples, while the testing produced secondary cracks at the left and right ends. This was because the test included multiple impacts, while in the simulation, the load was realized in one impact. Therefore, in an actual project, when dynamic load occurs, attention should be paid to protecting the top and bottom ends of the tunnel as well as the corners, and inspection of these locations should be strengthened.

#### 3.2.3. Failure Modes

The energy in a tunnel accumulates continuously under dynamic load conditions, and upon reaching a critical threshold, this energy is discharged, manifesting as cracks. Consequently, the failure of a tunnel is often closely linked to the fracture patterns in the surrounding rock. In this study, because the RHT material model is unable to distinguish between tensile and compressive damage, the displacement trend line method was used to confirm the failure mode of the samples. This approach, introduced by Zhang and Wong [37], summarizes the macroscopic failure behavior of the material into three basic types: tensile failure, mixed tensile–shear failure, and shear failure. In the tensile failure mode, the displacement trend lines are separated from each other, indicating that the cracks are mainly characterized by tensile failure, while in the shear failure mode, the displacement trend lines are close to each other, representing a strong shear effect. Moreover, displacement trend lines may show both separation and convergence, and the failure mode of the crack is then defined as mixed tensile–shear failure.

**Figure 15:**
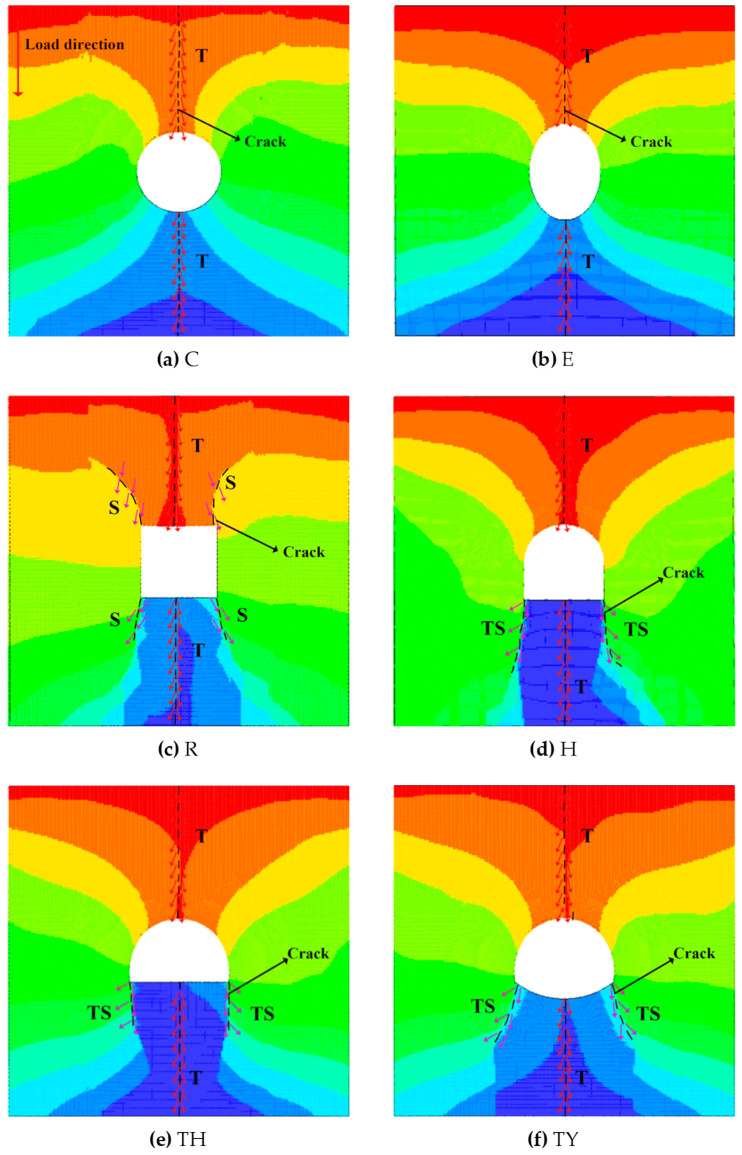
Displacement trend line of different shapes of model tunnels.

**Figure 16:**
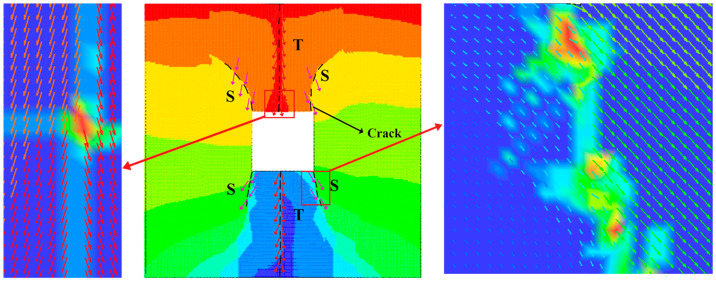
Localized enlargement of displacement line trend in the rectangular tunnel.

The results of the displacement trend lines for the different simulated tunnels are shown in Figure 15. To show more clearly the pattern in the displacement trend line, the direction of the displacement trend line at particular locations is marked with arrows in Figure 15, where T is a tensile failure, TS is a tension-shear failure, and S is a shear failure. In all the tunnels, the cracks produced at the top and bottom ends of the tunnels were determined from the displacement trend lines to be tensile failure. In Figure 16, the magnified visualization of the rectangular tunnel’s displacement trend line shows that the cracks at the top and bottom conform to the tensile failure displacement trend line and therefore represent tensile failure. The displacement trend line at the four corners matches shear failure displacement trend line and indicates shear failure. The cracks produced at the two corners in the lower regions of the horseshoe-shaped tunnel and the three-centered circular tunnel were mixed tensile–shear cracks. In summary, the failure of the samples was caused by the combined action of tensile and shear cracks, and its failure mode can be categorized as tensile-dominated failure.

#### 3.2.4. The Evolution Law of Dynamic Stress Concentration Factor

**Figure 17:**
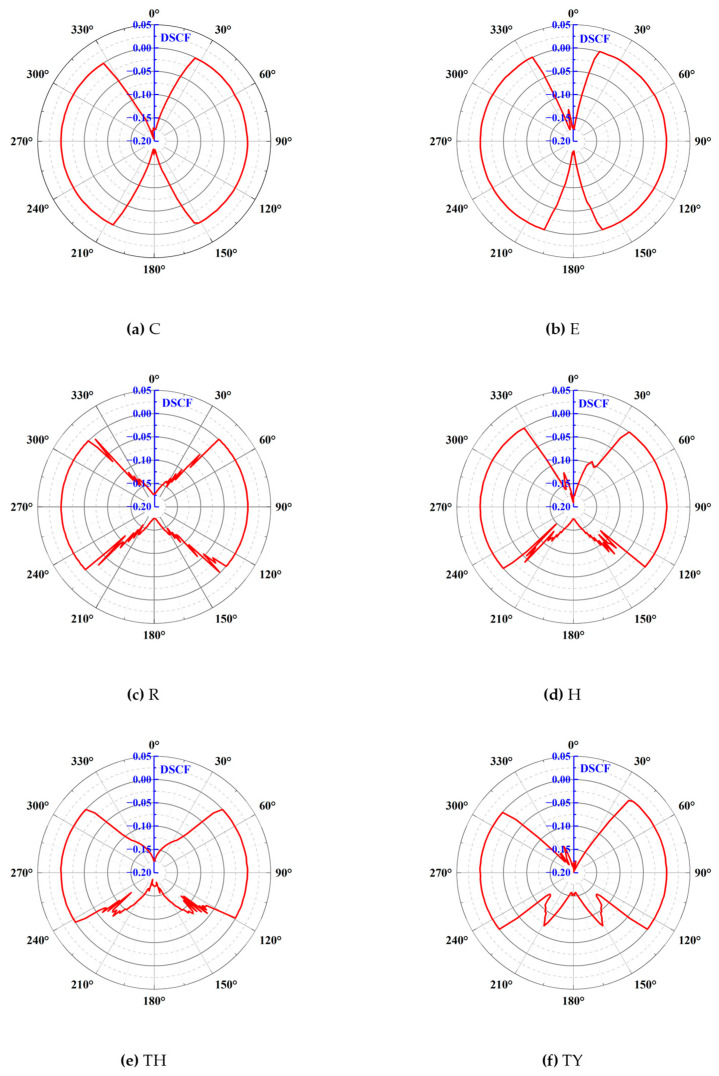
DSCF in the surrounding rock of different shapes of tunnels.

The results are presented in Figure 17. Because the structures of the tunnel model samples were symmetrical and the force was symmetrical, the distribution of DSCF was symmetrical. Only the stress concentration distribution in the range of 0° to 180° for the polar angle θ is discussed here. In the circular tunnel, the tensile stresses appeared in the ranges of 0° to 30° and 155° to 180° and gradually increased toward the top and bottom ends. The maximum DSCF that appeared at the top end was −0.194. The DSCF graph of the elliptical tunnel was similar in shape to that of the circular tunnel, but the tensile range at the top and bottom ends of the ellipse was reduced, with tensile stresses occurring only from 0° to 15° and from 165° to 180°, and a maximum DSCF of −0.179, which was a 7.7% reduction relative to that of the circular tunnel. Thus, it was observed that the elliptical tunnel alleviated the stress concentrations at the top and bottom ends to a certain degree when subjected to the force on its short axis, in accordance with the results in Section 3.1.1. The rectangular tunnel started to show tensile stress concentration from the four corners, and the DSCF gradually increased from the corners to the top and bottom ends. The horseshoe-shaped tunnel had a smaller tensile range compared with the top end of the rectangular tunnel, with a maximum DSCF of −0.179. The graph for the three-centered circular tunnel was similar to that for the horseshoe-shaped tunnel, with tensile stress concentrations at the top and bottom ends. Moreover, the DSCFs of the horseshoe-shaped tunnel, three-centered circular tunnel, and three-centered circular tunnel with the inverted arch all showed varying degrees of stagnation or rebound phases at the bottom end, ranging from 10° to 15°. This indicates that the upper arch also had a cushioning action on the stress concentrations in the lower part. The inverted arch relieved the stress concentration at the bottom of the three-centered circular tunnel, to a certain extent. Firstly, the tensile range of the three-centered circular tunnel with inverted arch was lower than that of the three-centered circular tunnel. Secondly, the DCSF at the bottom of the three-centered circular tunnel was −0.185, while that of the three-centered circular tunnel with the inverted arch was −0.159, representing a drop of 14.1%.

#### 3.2.5. Dynamic Stress Concentration Factor Under Lateral Pressure

**Figure 18:**
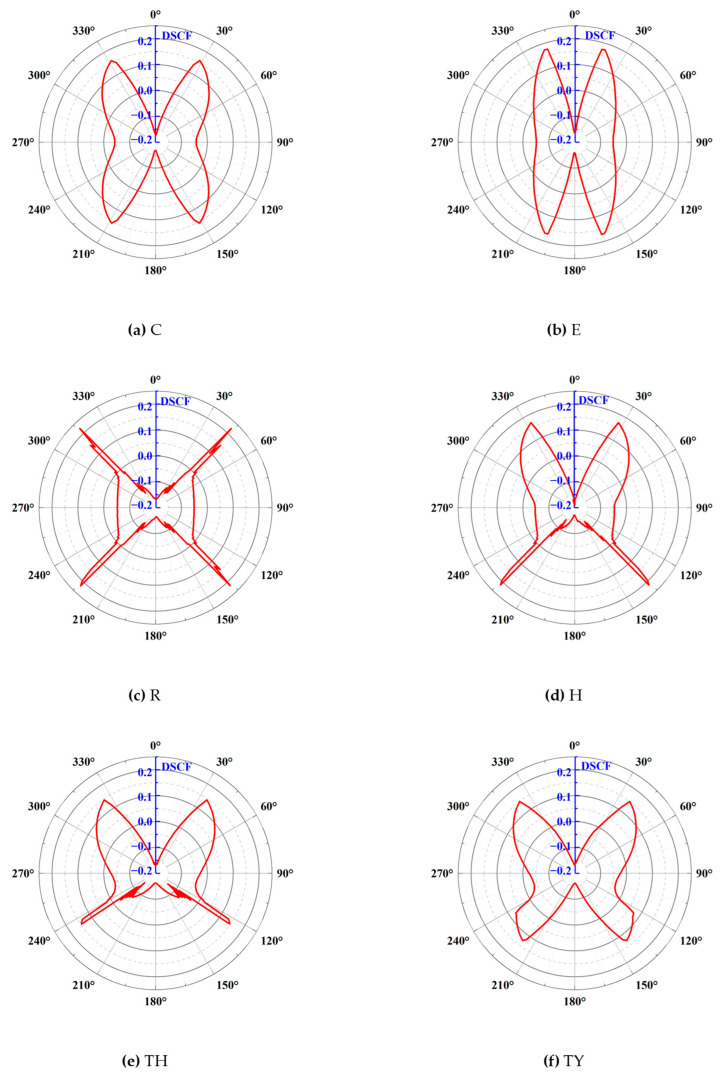
DSCF of different tunnel shapes under lateral pressure.

The dynamic mechanical properties of rocks under the action of in situ stress have been extensively studied by various methods such as numerical simulations and experiments [38,39,40,41]. The combined effect of dynamic load and pre-stress can cause the surrounding rock to be damaged. Based on the simulation mentioned above, the ground stress conditions deep underground were simulated by applying 2 MPa lateral pressure. The DSCFs of the tunnels with different shapes are further discussed, and the results are shown in Figure 18. Compared with Figure 17, it can be seen that the DSCFs of all tunnels decreased at the top and bottom ends after adding lateral pressure, while the DSCF at the corners was positive, indicating that these regions were under a state of concentrated compressive stress. The DSCF graphs of the circular and elliptical tunnels are similar in shape. The tensile stress concentration factor at the top and bottom ends of the circular tunnel was larger than in the elliptical tunnel, but the maximum DSCF of 0.158 for the circular tunnel was smaller than that of the elliptical tunnel with a maximum DSCF of 0.175, which was due to the elliptical long axis being under pressure, which is liable to lead to stress concentration. The rectangular tunnel produced compressive stress concentration factors at the four corners, and 0.223 was the maximum for all the tunnels. The horseshoe-shaped tunnel consisted of a combination of a circular upper portion and a rectangular lower portion. The three-centered circular tunnel had a similar shape to the horseshoe-shaped tunnel, but the tensile stress concentration factor and the compressive stress concentration factor were smaller than those of the horseshoe-shaped tunnel. Finally, the compressive stress concentration factor at the lower corners of the three-centered circular tunnel with the inverted arch was less than that of the three-centered circular tunnel, also reflecting the cushioning action of the inverted arch.

**Figure 19:**
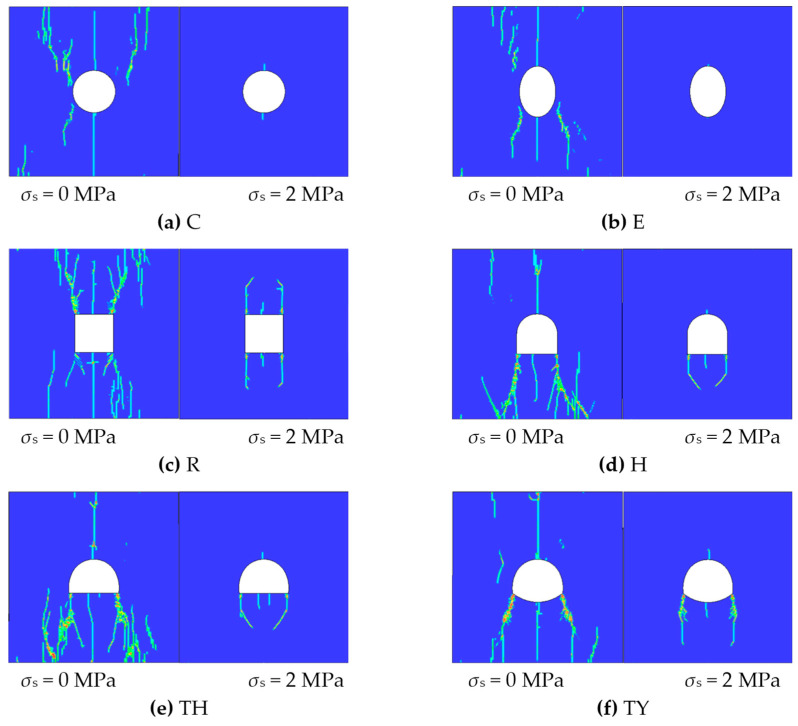
Comparison of cracks.

After adding lateral pressure, failure modes at 1100 μs were selected for comparison with those without lateral pressure, as shown in Figure 19. The lateral pressure had a certain inhibitory effect on the fracture behavior, and the length and width of the cracks were much smaller than those without the lateral pressure. Moreover, secondary cracks did not occur under lateral pressure. At a lateral pressure of 2 MPa, the failure of the elliptical tunnel was clearly the least severe, again showing that the stability of the elliptical tunnel was the best compared with the other tunnels.

## 4. Summary and Conclusions

This study provides new insights into how tunnel geometry influences dynamic mechanical properties and failure mechanisms. Through quantitative analysis of stress concentration factors and energy dissipation trends across different tunnel geometries, it emphasizes the importance of optimizing tunnel section shapes and improving inverted arch designs to enhance resistance to dynamic loads, thereby improving the structural safety and durability of tunnels in practical engineering applications. The results of this study are summarized as follows:

(1) In the circular and elliptical tunnels and in the three-centered circular tunnel with an inverted arch, the main tensile cracks were mainly generated at the top and bottom ends of the tunnels. In addition to the main tensile cracks at the upper and lower ends of the rectangular tunnel, random shear cracks appeared at the four corners. The horseshoe-shaped tunnel and the three-centered circular tunnel were associated with tensile cracks at the top, and the two corners experienced mixed tensile shear cracking.
(2) The tunnels’ shapes significantly affected the dynamic compressive strength of the samples. The magnitude of sample strength followed the order E > C > H > TY > TH > R. The stability of the elliptical tunnel was the best under the condition of the same cross-section area. Therefore, in some real projects, the elliptical section can be preferred as the tunnel section, and the rectangular section can be avoided as far as possible.
(3) The dissipated energy percentage of the samples were calculated to characterize the degree of destruction, based on the relationship between incident, reflected, and transmitted energy. It is concluded that the dissipative energy proportion of the three-centered circular tunnel was the largest, and that of the elliptical tunnel was the smallest. From an engineering perspective, since three-centered circular tunnels absorb the most energy, their structural integrity should be closely monitored after dynamic disturbances, to ensure safety and stability.
(4) Regardless of the shape of the tunnel, the maximum tensile stress concentration factor appeared at the bottom or top of the sample; so, the locations of the main crack sprouting were most commonly at the bottoms and tops of the tunnels. However, in the tunnels that included corners, the cracks also propagated outward from these corners even though the maximum tensile stress concentration was not attained there. Therefore, in a practical project, reinforcing the support at the top, bottom, and corners of the tunnel is essential.
(5) The inverted arch is one of the main components of the tunnel structure. The inverted arch can significantly alleviate the stress concentration phenomenon in the bottom corners, which affects crack propagation. Therefore, in an actual project, the aim of an inverted arch is to increase the support resistance for the lower regions and walls to prevent shear failure caused by internal crowding.

Overall, this study reveals the failure mechanisms of tunnels with different shapes under dynamic loading, providing a theoretical basis for tunnel design optimization, particularly for tunnel inspection and maintenance following impact loads. The findings have significant practical applications in geotechnical engineering and tunnel construction. This study used green sandstone as the representative material for the tunnel rock mass, which brings certain limitations, as real tunnel rock masses may be found not only in green sandstone but also in harder rock strata. Additionally, real tunnel rock masses often exhibit anisotropy and are present in more complex environments with varying bedding and fracture structures. Nevertheless, the choice of green sandstone as the experimental material is rational, as its mechanical properties are relatively uniform, providing a simplified experimental model and ensuring the feasibility of the experiment. Future research should involve conducting experiments on a wider variety of rock materials, including different rock types and more complex rock mass structures.

## Figures and Tables

**Table 1:** Physical and mechanical parameters of green sandstone.

Parameter	Value
Density (kg/m^3^)	2249
Elastic modulus (GPa)	13.47
Poisson’s ratio	0.21
Compressive strength (MPa)	24.25
Tensile strength (MPa)	2.08
P-wave velocity (m/s)	2543
S-wave velocity (m/s)	1669

**Table 2:** RHT model parameters for green sandstone.

Parameter	Value
Density (kg/m^3^)	2249
Elastic shear modulus (GPa)	5.579
Relative shear strength	0.18
Relative tensile strength	0.089
Residual surface parameter, AF	1.6
Residual surface parameter, NF	0.61
Parameter for polynomial EOS, B_0_	1.22
Parameter for polynomial EOS, B_1_	1.22
Reference compressive strain rate	3 × 10^−5^
Reference tensile strain rate	3 × 10^−6^
Break compressive strain rate	3 × 10^25^
Break tensile strain rate	3 × 10^25^
Compressive yield surface parameter	0.51
Crush pressure (MPa)	16.1
Compaction pressure (GPa)	6
Eroding plastic strain	2
Minimum damaged residual strain	0.01
Tensile strain rate dependence exponent	0.045
Compressive strength (MPa)	24.25
Parameter for polynomial EOS, T_1_ (GPa)	13.37
Parameter for polynomial EOS, T_2_ (GPa)	0
Damage parameter, D_1_	0.04
Damage parameter, D_2_	1
Hugoniot polynomial coefficient, A_1_ (GPa)	13.37
Hugoniot polynomial coefficient, A_2_ (GPa)	19.68
Hugoniot polynomial coefficient, A_3_ (GPa)	8.545
Failure surface parameter, A	1.6
Failure surface parameter, N	0.61
Lode angle dependence factor, Q_0_	0.6805
Lode angle dependence factor, B	0.0105
Tensile yield surface parameter	0.7
Shear modulus reduction factor	0.5
Porosity exponent	3
Initial porosity	1
Pressure influence on plastic flow in tension	0.001
Compressive strain rate dependence exponent	0.43
Gruneisen gamma	0

## Data Availability

The original contributions presented in this study are included in the article. Further inquiries can be directed to the corresponding authors.

## Abbreviations

The following abbreviations are used in this manuscript:

SHPB	Split Hopkinson pressure bar
DIC	Digital image correlation
C	Circular tunnel
E	Elliptical tunnel
R	Rectangular tunnel
H	Horseshoe-shaped tunnel
TH	Three-centered circular tunnel
TY	Three-centered circular tunnel with inverted arch
RHT	Riedel–Hiermaier–Thoma
DSCF	Dynamic stress concentration factor
